# Implications of estrogen and its receptors in colorectal carcinoma

**DOI:** 10.1002/cam4.5242

**Published:** 2022-10-07

**Authors:** Plabon Kumar Das, Joti Saha, Suja Pillai, Alfred K.‐Y. Lam, Vinod Gopalan, Farhadul Islam

**Affiliations:** ^1^ Department of Biochemistry and Molecular Biology University of Rajshahi Rajshahi Bangladesh; ^2^ Institute for Glycomics Griffith University Gold Coast Queensland Australia; ^3^ Department of Applied Chemistry and Chemical Engineering University of Rajshahi Rajshahi Bangladesh; ^4^ School of Biomedical Sciences, Faculty of Medicine The University of Queensland Brisbane Queensland Australia; ^5^ School of Medicine & Dentistry Griffith University Gold Coast Queensland Australia

**Keywords:** chemotherapy, targeted therapy, colorectal carcinoma, estrogen, estrogen receptors

## Abstract

Estrogens have been implicated in the pathogenesis of various cancer types, including colorectal carcinoma (CRC). Estrogen receptors such as ERα and ERβ activate intracellular signaling cascades followed by binding to estrogen, resulting in important changes in cellular behaviors. The nuclear estrogen receptors, i.e*.* ERβ and ERα are responsible for the genomic actions of estrogens, whereas the other receptor, such as G protein‐coupled estrogen receptor (GPER) regulates rapid non‐genomic actions, which lead to secondary gene expression changes in cells. ERβ, the predominant estrogen receptor expressed in both normal and non‐malignant colonic epithelium, has protective roles in colon carcinogenesis. ERβ may exert the anti‐tumor effect through selective activation of pro‐apoptotic signaling, increasing DNA repair, inhibiting expression of oncogenes, regulating cell cycle progression, and also by changing the micro‐RNA pool and DNA‐methylation. Thus, a better understanding of the underlying mechanisms of estrogen and its receptors in CRC pathogenesis could provide a new horizon for effective therapeutic development. Furthermore, using synthetic or natural compounds as ER agonists may induce estrogen‐mediated anti‐cancer activities against colon cancer. In this study, we report the most recent pre‐clinical and experimental evidences related to ERs in CRC development. Also, we reviewed the actions of naturally occurring and synthetic compounds, which have a protective role against CRC development by acting as ER agonist.

## Graphical abstract

ERß, the predominant estrogen receptor expressed in both normal and non‐malignant colonic epithelium, has a protective role in colon carcinogenesis. ERß may exert the anti‐tumor effect through selective activation of pro‐apoptotic signaling, increasing DNA repair, inhibiting expression of oncogenes, regulating cell cycle progression, and also by changing the micro‐RNA pool and DNA‐methylation.

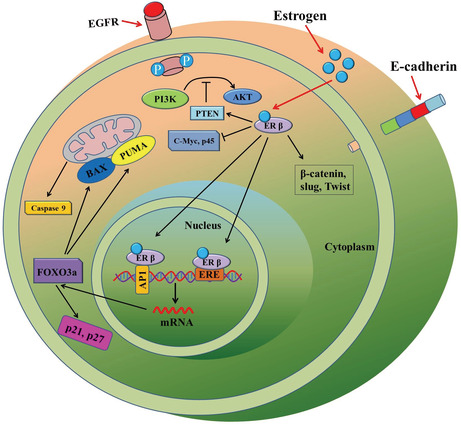

## 1 INTRODUCTION

Estrogens are known to play essential roles in the initiation and progression of multiple tumor types, especially tumors of hormonally regulated tissues such as the breast, endometrium and ovary.^1^, ^2^ In general, estrogen acts as a cancer‐protective hormone through its anti‐inflammatory activities; however, the roles of individual estrogen (i.e. nuclear or membrane) receptors are often controversial in colorectal cancer (CRC) genesis.^2^ The functionality of estrogens in cancer is mediated by the nuclear estrogen receptors α and β (ERα and ERβ), as well as by the membrane‐bound G protein‐coupled estrogen receptor (GPER).^1^ ERβ, the most prominent among all the estrogen receptor, acts as a tumor suppressor in CRC along with prognostic significance.^3^ Expression of ERβ in the intestinal crypts may protect the body from developing cancer.^4^ Thus, downregulation of the ERβ receptor in intestinal mucosa may result in the development of CRC.^3^, ^4^ A significantly lower level of ERβ was noted in CRC patients with advanced clinical stages (stages III & IV) when compared with early stages (stages I & II) patients. Moreover, deficiency of estrogen receptor α (ERα) along with ERβ modulates colon neoplastic transformation and abnormal mucosal formation in an APC‐dependent tumourigenesis model.^3^ Furthermore, GPER can positively regulates cell proliferation, migration and invasion of colon cancer cells, thus, promoting the pathogenesis and progression of CRC.^1^, ^5^ By cross talking with other signaling pathways such as RAS/RAF/ERK1/2 and EGFR pathways, GPER may also regulate colonic mobility.^6^ Estrogens and their receptors show either positive or negative roles in the pathogenesis of CRC. A deep understanding of the underlying molecular mechanisms of estrogen and its receptors may provide prospects for improved CRC management. In this review, we discuss the roles of estrogen receptors and their multiple molecular targets in CRC pathogenesis. We also outlined the use of some potential natural or synthetic compounds to target ER‐mediated signaling in colon cancer cells to inhibit their proliferation and progression.

## 2 ROLE OF ESTROGEN RECEPTORS (ERS) IN CRC PATHOGENESIS

### 2.1 The expression characteristics of ERβ and ERα

Estrogen signaling is mediated primarily by ERα and ERβ, which are encoded by *estrogen receptor 1*(*ESR1*) and *estrogen receptor 2* (*ESR2*) genes, respectively.^7^, ^8^ Estrogen, with its receptors ERβ and ERα regulates genes transcription through genomic and non‐genomic responses via activating rapid signal transduction pathways.^9^, ^10^ Genomic effects of estrogen in CRC begin with an activated ER interacting with either specific DNA sequences, also known as estrogen response elements (ERE) or other transcription factors, including c‐Jun and c‐Fos.^11^ Though the interaction of both ERs (ERβ and ERα) with ERE is quite similar, however, ERα interacts with c‐Jun and c‐Fos of the activating protein‐1 complex (AP‐1) and transcription factor specificity protein 1 (SP1) more frequently than ERβ. In the case of AP1, binding of estrogen with ERα results in activation of transcription, whereas binding of estrogen with ERβ suppresses transcription by ablation of estrogen from the ERα pathway.^12^, ^13^ Thus, the predominant ERβ expression switch‐off ERα meditated activation of downstream signaling by competitive inhibition. However, ERs can also be phosphorylated and activated by the activated kinase pathway without even binding to estrogen ligand. For example, EGFR can activate the Ras/Raf/MAPK pathway, which simultaneously phosphorylates ERs, thereby resulting in dimerization and ligand‐independent activation of target genes expression.^14^, ^15^

### 2.2 Mechanism of actions of ERβ and ERα in CRC pathogenesis

The non‐genomic actions of estrogen induced‐cell proliferation are triggered by the localization of ERs at the plasma membrane.^10^ Palmitoylation of ERα allows them to localize at the plasma membrane and then to interact with caveolin‐1.^10^ This interaction results in the activation of a rapid signaling cascade associated with cell proliferation upon hormonal stimulation. However, ERβ palmitoylation exhibits opposing effects and minimizes ERα induced changes in cellular physiology. ERβ acts as a substrate for palmitoyl acyltransferase (PAT). This palmitoylation of ERβ allows localizing at the plasma membrane and interacts with caveolin‐1. Upon this interaction, unlike ERα, the hormonal stimulation causes increased interaction of ERβ with caveolin‐1 and p38 member of the MAPK family. These signaling cascades help estrogen to mediate its anti‐proliferative effect in CRC cells by increasing pro‐apoptotic signals.^10^ In addition, co‐expression of ERα with ERβ resulted in a concentration‐dependent reduction of ERα‐mediated transcription, indicating ERβ as the dominant regulator of estrogen signaling.^16^, ^17^ ERβ induced growth reduction of colon cancer cells and frequent loss of expression in clinical samples indicated the tumor protective feature of ERβ in colon cancer.^12^, ^18^, ^19^, ^20^ Table 1 shows the genes or signaling pathways regulated by ERs in CRC pathogenesis.

**TABLE 1:** Estrogen receptors and their functions in colon cancer

Receptors	Targets	Functions	References
ERβ	hMLH1	Estrogen binding to ERβ can increase the expression of mismatch repair gene *hMLH1* to inhibit cancer cells proliferation	[21]
	p21 and p27	Upregulation of p21 and p27 proteins activate caspase‐8 and caspase‐9 to induce apoptosis of cancer cells	[22]
	p53 signaling	Causes up‐regulation of pro‐apoptotic p53 target genes *Bax*, *Noxa*, and *PUMA*	**[** 23 **]**
	miR‐17	Inhibits proliferation of cancer cells by down regulation of oncogene *Myc*	[24]
	miR‐205	Inhibits the expression of PROX1, which subsequently reduces the metastatic potential of cancer cells	[25]
	Cyclin E p21CIP1	Inhibits proliferation by modulating cell cycle components, which subsequently results in G1‐S cell cycle arrest	[26
	p45Skp2	Causes inhibition of cellular proliferation by downregulation of cdk2‐inhibitor p27Kip1 at posttranscriptional level	[27]
	CyclinD1	Inhibits the proliferation of cancer cells by downregulating the expression of CyclinD1	[27]
	mTOR BNIP3	Suppresses the expression of mammalian target of rapamycin (mTOR) or activates Bcl‐2/adenovirus E1B 19‐kDa‐interacting protein 3 (BNIP3) to promote autophagy in cancer cells	[27]
GPER	Bcl‐2‐associated X protein, p21, and cleaved caspase‐3, Bcl‐2, procaspase‐3	Induces apoptosis of cancer cells by increasing the expression of Bcl‐2‐associated X protein, p21, and cleaved caspase‐3, and by decreasing the expression of anti‐apoptotic factors Bcl‐2 and procaspase‐3	[6]
	Activating transcription factor 6 (ATF6), X box binding protein 1 (XBP‐1) and C/EBP‐homologous protein (CHOP)	Increases expression of ATF 4 and 6, X box binding protein 1 (XBP‐1) and C/EBP‐homologous protein (CHOP)to induce endoplasmic reticulum stress mediated growth arrest and apoptosis in cancer cells	[28]
	Reactive oxygen species (ROS), ERK1/2	Increases the expressions of ROS and also increases ERK1/2 phosphorylation, which results in growth arrest of cancer cells	[28]
	Fatty acid synthase (FASN)	Increases the expression and activity of FASN through EGFR/ERK/c‐Fos/AP‐1 signaling, which subsequently results in increased growth and migration of cancercells	[29]

**FIGURE 1:**
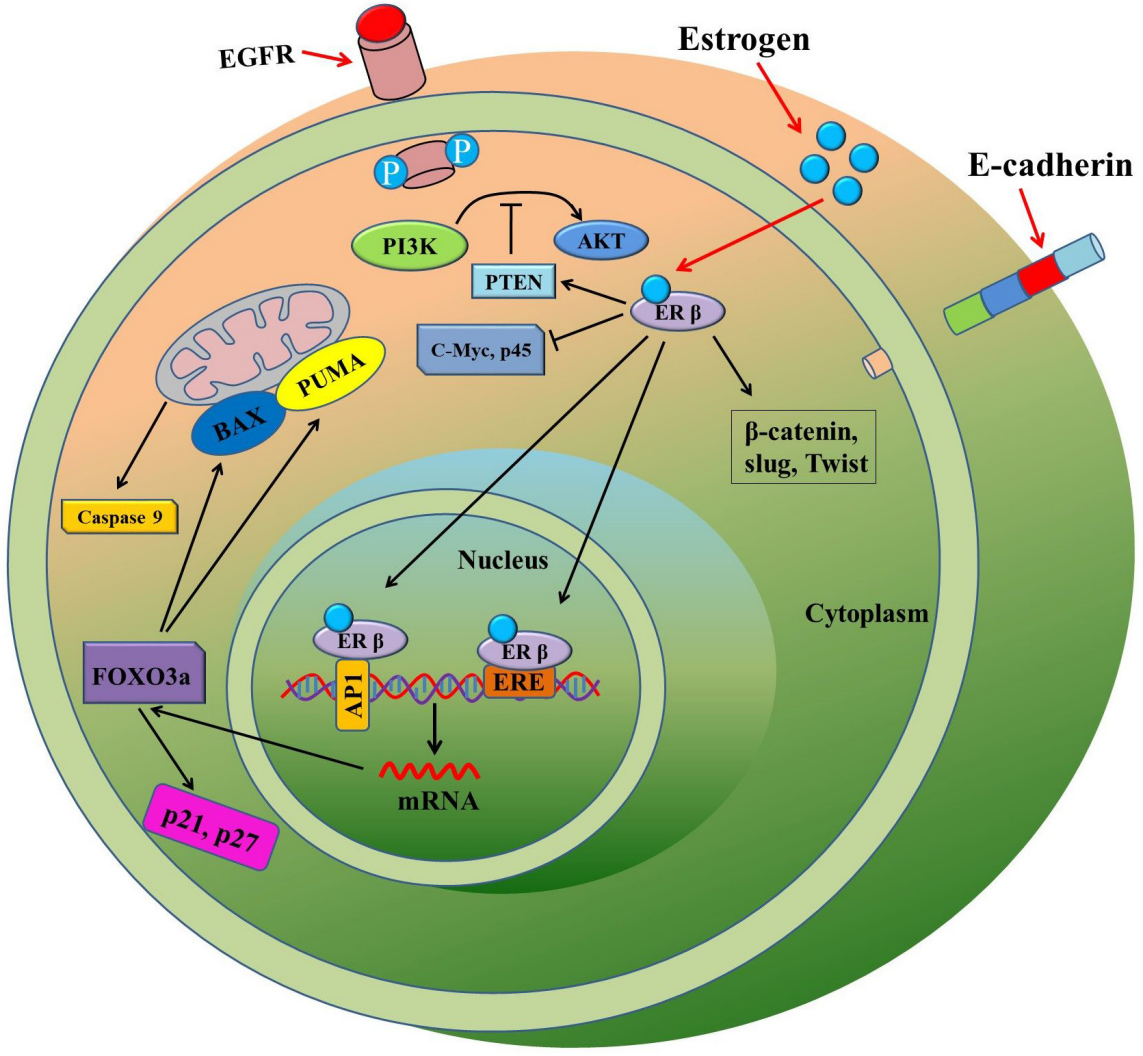
Roles of ERβin CRC pathogenesis. Upon binding to its ligand ERβ becomes activated and transcriptionally upregulates downstream target genes such as FOXO3a. The target genes carry ERβ binding elements such as ERE or AP1. The activated FOXO3a in turn transcriptionally upregulates PUMA, p21, and p27. ERβ also inhibits expression of genes such as *c‐Myc* and *p45Skp2*. Moreover, some of the EMT and metastasis genes, such as *β‐catenin*, *Slug* and *Twist*, are inhibited by ERβ.

In the normal colon, ERβ plays vital roles in maintaining the epithelial architecture, regulating gastrointestinal physiology and in mediating immune responses.^30^ Reduced ERβ expression has been associated with gut permeability, and most importantly, with colitis, a risk factor for CRC development.^31^ However, there have been limited number of studies on the differential expression of ERα and ERβ in both matched non‐neoplastic and cancerous tissues. A study noted that the mRNA expression of ERβ was higher in the normal colonic epithelium at 91.7% than 83.3% in CRC tissue. Whereas the expression of ERα mRNA was lower at 16.6% in non‐cancerous colonic epithelium than in CRC tissues, which was 25%.^32^ An in‐vivo study by Wada‐Hiraike et al. suggested an increase in epithelial cell proliferation and reduced apoptosis was noted in ERβ knockout mice^33^ in comparison to that of control animals. A compartmentalization study of ERα and ERβ expression within the crypt axis suggested that ERβ negatively regulates the expression of ERα as colonic enterocytes differentiate and undergo growth arrest.^3^ Increased ERβ expression in enterocytes of the ascending crypts causes higher competition with ERα for its ligand, as well as for co‐activators, which may restrain ERα actions.^3^ Also, increased ERβ expression could lead to ERα‐ERβ hetero‐dimerization, thereby the expression pattern of target genes may alter as in proliferating cells, ERα abundance favors its homo‐dimerization.^3^ This phenomenon reveals an important balance kept in the expression of each ER subtypes and their co‐expressions in the cells.^10^ A molecular mechanism of ERβ actions has been illustrated in Figure 1. As ERβ is the predominant estrogen receptor and it suppresses ERα activity, therefore this review will focus on the actions of ERβ, along with GPER.

### 2.3 Roles of ERβ in CRC

#### 2.3.1 ERβ mediated DNA repair

ERβ manifests its anti‐carcinogenesis properties in human cells in several ways, firstly via induction of prompt DNA repair capacity and, secondly, by increasing apoptosis of cancer/transformed cells.^21^, ^34^ Jin et al. found a positive correlation between the increased concentration of serum estrogen level in human colonic epithelial cells and upregulation of DNA mismatch repair gene (*hMLH1*) expression.^21^ Estrogen induced overexpression of hMLH1 was reversed in ERβ‐positive colon cancer cells upon the supplementation of estrogen antagonist (ICI182.780). For example, estrogen‐induced hMLH1 overexpression was noted in ERβ‐positive COLO205 colon cancer cells, which was later downregulated by estrogen antagonist (ICI182.780) supplementation.^21^ These findings implied that the anti‐colonic cancer effect of estrogen might be related to the regulation of *hMLH1*, suggesting estrogen‐mediated DNA repair in cancer cells.

#### 2.3.2 ERβ mediated induction of apoptosis of colon cancer cell

ERβ instigates apoptosis of colon cancer (LoVo) cells by activating p53 (TP53) signaling.^22^ Estrogen and ERβ induce upregulation of p53 downstream components p21 and p27, which activates caspase‐8 and caspase‐9, resulting in induction of apoptosis of cancer cells. In addition, overexpression of estrogen and ERβ resulted in downregulation of β‐catenin proteins, which subsequently cause the downregulation of its target genes (cyclin D1 and Rb), thereby suppressing cell growth and proliferation non‐malignant colonocytes.^23^ Furthermore, estrogen‐mediated activation of p53 signaling causes up‐regulation of pro‐apoptotic p53 target genes, including *Bax*, *Noxa*, and *PUMA*, which ultimately switch on the apoptosis of cancer cells.^23^

#### 2.3.3 ERβ mediated inhibition of oncogenes in CRC

**FIGURE 2:**
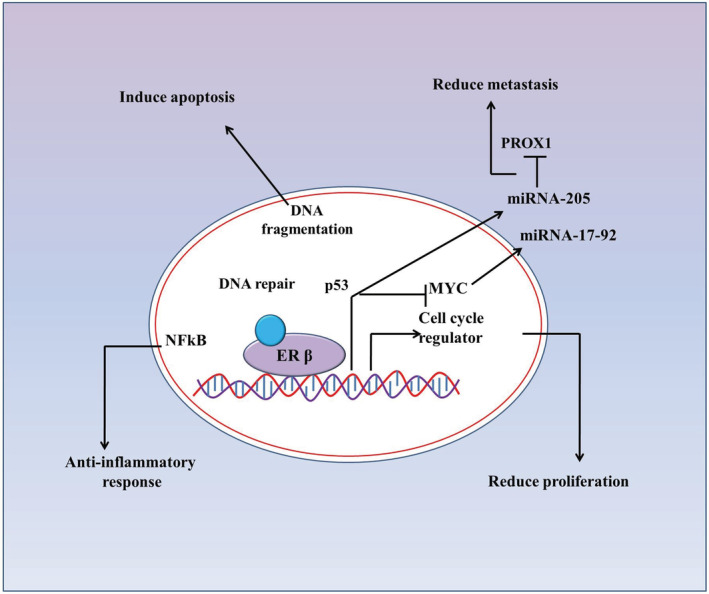
Molecular mechanism for ERβ‐mediated anti‐tumorigenic activity in CRC. ERβ mediates its anti‐tumourigenic property by inducing prompt DNA repair capacity, increasing apoptosis signaling, enhancing anti‐inflammatory response and by reducing tumor metastasis.

Additionally, the anti‐tumourigenic effect of ERβ in cancer cells could be a combination of downregulation of oncogenes such as *PROX1*, *Myc*, and *MYB* along with increased DNA repair capacity and induction of apoptosis (Figure 2). Expression of ERβ changes micro‐RNAs (miRs) pool in colon cancer cells, which in turn, could downregulate the expression of *Myc* and *Prospero homeobox protein‐1* (*PROX1*) oncogenes.^24^, ^35^, ^36^ Petrova et al. reported that PROX1 drives the transition from benign adenoma to carcinoma, and silencing of this transcription factor resulted in inhibition of human colorectal tumor growth in vivo.^37^ It was noted that PROX1downregulation was associated with CRC progression, poor tumor differentiation and low patient survival.^36^ Further study indicated that ERβ induced PROX1 inhibition via miR‐205 expression. Inhibition of PROX1 expression caused reduced metastatic potential of colon cancer cells in vivo.^25^ Thus, a signaling cascade of ERβ, miR‐205 and PROX1in the colorectum may confer protective roles against carcinogenesis. Therefore, it is hypothesized that ERβ protects against tumorigenesis in CRC by enhancing DNA repair and apoptosis while repressing oncogene expression, thereby proliferation and metastasis.

#### 2.3.4 ERβ mediated cell cycle regulation in CRCs

Expression of ERβ could induce altered cell cycle kinetics by regulating the expression of several components, including cyclin E, cyclin D1 etc., in colon cancer cells.^26^, ^27^, ^38^ ERβ mediated altered expression of these components causes cell cycle arrest, resulting in reduced proliferation of cancer cells.^26^ For example, Hartman et al. reported that ERβ over‐expression inhibits the proliferation of colon cancer HCT8 cells by decreasing cyclin E expression and by increasing CDK inhibitor p21CIP1 expression. This ERβ mediated changes of cyclin E and CDK inhibitor p21CIP1 causes G1‐S phase cell cycle arrest in HCT8 cells.^26^ Also, a significant reduction of in vivo tumor volume was seen, followed by transplantation of ERβ overexpressing cells.^26^ Another study noted that c‐Myc, a well‐known oncogene and commonly overexpressed in a majority of CRCs, was downregulated in the ERβ‐expressing cancer cells.^27^ Furthermore, Wei et al. reported that ERβ played an anti‐proliferative role in HCT116 colon cancer cells by impairing the cell cycle but not apoptosis, through downregulating the expression of CyclinD1.^27^ In CRC, the expression of CyclinD1 was increased significantly when compared with normal colon tissue. However, ERβ overexpression induced CyclinD1 downregulation significantly. The study further confirmed that ERβ mediated inhibition of cell proliferation was not by apoptosis as there was no statistically significant difference in the expression of pro‐Caspase‐3 and cleaved‐Caspase‐3 between the control group and the group with higher expression of ERβ.^27^ Besides, ERβ could inhibit the mammalian target of rapamycin (mTOR) or activate Bcl‐2/adenovirus E1B 19‐kDa‐interacting protein 3 (BNIP3) to promote autophagy in colon cancer (HCT116) cells.^27^ These results indicate that ERβ‐mediated CyclinD1 degradation in the combination of mTOR/BNIP3 deregulation could induce inhibition of cancer cell growth through induction of autophagy followed by cell cycle arrest. Therefore, loss of ERβ expression could be one of the events involved in the development or progression of colon cancer. Thus, devise of approaches or mechanism which could lead the expression of ERβ or keep ERβ expression in colonic epithelium might provide a preventive strategies in the management of patients with CRC in clinical settings.

#### 2.3.5 ERβ‐mediated epigenetic regulation of CRCs

miRNA mediated regulations of genes expression play a vital role in proliferation, apoptosis and migration of colon cancer cells.^39^, ^40^, ^41^, ^42^ Previous studies have reported that ERβ induced alteration of miRs in colon cancer (Table 2), resulting in changes of miRs mediated cellular pathophysiology, which in turn could provide cancer‐protective features.^26^, ^35^, ^36^ In recent studies, the expressions of 27 miRNAs were changed following ERβ expression in CRC and most of these miRNAs are implicated in the pathogenesis of gastrointestinal cancers.^47^, ^48^, ^49^ Also, many of the mRNA targets of these miRs were concomitantly changed upon ERβ expression. For example, Hur et al. found that miR‐200 was strongly downregulated upon ERβ expression, which resulted in increased ZEB1 and decreased E‐cadherin expressions. In mammary epithelial cells, restoration of miR‐200 expression has been found to inhibit the migration, whereas its downregulation was associated with increased migration in colon cancer cells.^43^ In addition, supplementation of miR‐200c mimics causes increased Myc and its target genes expression, which in turn mediated the progression of CRC.^43^ ERβ‐mediated downregulation of *Myc* was further enhanced by concomitant downregulation of miR‐200a/b.

**TABLE 2:** ER mediated changes in miRNA pool in CRC

MicroRNA	Expression pattern in CRC	Associated functions	References
miR‐205	Downregulated	Inhibits the expression of PROX1, which subsequently reduces the metastatic potential of CRC cells	[25]
miR‐200a/b	Downregulated	Decreases ZEB1 and increases E‐cadherin expressions	[43]
miR‐17	Downregulated	Increases the expression of *NCOA3* and *CLU*, which subsequently decreases metastasis and tumor growth.	[35]
miR‐196a‐1	Upregulated	Inhibits Annexin A1 mediated anti‐tumourigenesis in cancer cells	[44]
miR‐19a	Upregulated	Induces tumourigenesis by inhibiting tumor suppressor gene *DLC1*	[45]
miR‐25	Upregulated	Induces tumourigenesis by inhibiting tumor suppressor gene *LATS2*	[46]

Another miRNA mi‐17 is involved in ERβ mediated signaling in colon cancer cells.^35^, ^50^ miR‐17 mediates the upregulation of *nuclear receptor co‐activator 3* (*NCOA3*) and *clusterin* (*CLU*) followed by ERβ expression. Inverse‐correlation between miR‐17 and its targets such as *NCOA3* and *CLU* was noted in CRC. miR‐17induced reduction of *CLU* and *NCOA3* resulted in enhanced angiogenesis and cell growth in colon cancer cells.^35^, ^50^

Annexin A1 (ANXA1) is another target of miRNA that are implicated in colon carcinogenesis. ANXA1 is a calcium‐dependent phospholipid‐linked protein that has been associated with drug resistance, regulation of cellular differentiation, proliferation and apoptosis.^44^ miR‐196a‐1 suppresses the anti‐tumourigenic activity of ANAX1 by inhibiting its expression. However, ERβ expression resulted in the downregulation of miR‐196a‐1, which in turn recovers the anti‐tumourigenic property of ANAX1. ANAX1 mediates its action by inhibiting nuclear factor‐κB and inflammatory response in the colon.^44^ Many tumor suppressors such as *Deleted in Liver* Cancer *1*(*DLC1)* and *Large tumor suppressor, homolog* 2 (*LATS2*), downregulated in colon cancer cells, are upregulated followed by ERβ expression and targeted by miRNAs. For instance, *DLC1*can inhibit proliferation in colon cancer cells, is a target of miR‐19a, whereas, tumor suppressor *LATS2* is targeted by miR‐25.^45^, ^46^ Thus, exploring the networks between ERβ‐affected miRNAs and their target genes is likely to contribute to elucidating the mechanism of transcriptional regulation by ERβ.

Additionally, expression of ERβ associated with DNA‐methylation in patients with CRC by binding to the target DNA, thereby modulate menopausal hormonal therapy, which in turn reduced the risk of CRC development.^51^ Also, epigenome‐wide analysis of DNA‐methylation revealed that ERβ expression associated with promoter methylations of genes involved in CRC pathogenesis.^52^ Furthermore, it was reported that moderate/high expressions of ERβ are associated with overall survival in patients with CRC.^52^ Altogether, discovery of ERβ raises hopes for developing better endocrine therapeutic options for patients with CRC, however, the effective therapeutic modalities targeting ERβ is yet to be established. One of the reason could be the antibodies used for ERβ expression analysis in clinical and preclinical studies are might be unspecific.^53^ Thus, uses of specific antibodies in preclinical, clinical and validation studies could provide better insight of clinical implication of ERβ expression in patients with CRC.

### 2.4 GPER in colon cancer cells

**FIGURE 3:**
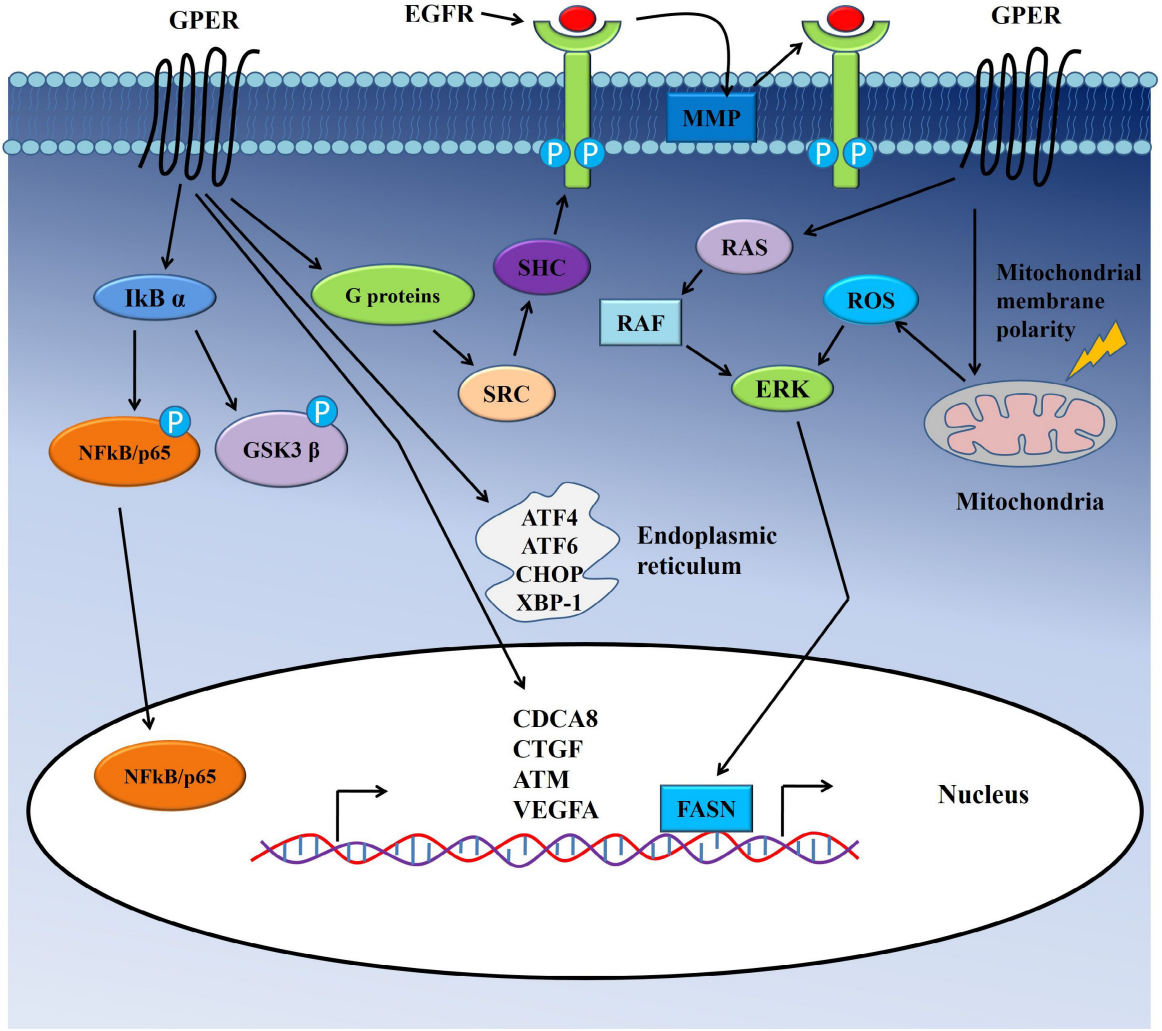
Signaling pathways modulated by G protein‐coupled estrogen receptor in colon cancer cells. Upon binding of ligands, GPER starts a signaling cascade which ultimately results in target genes expression to maintain various properties of colon cancer cells.

G protein‐coupled estrogen receptor (GPER) plays essential roles in carcinogenesis by regulating cancer cell growth and survival by a selective interaction with ligands G‐1(Figure 3).^54^ GPER associated with the regulation of proliferation and survival not only in estrogen‐associated cancers such as breast,^54^, ^55^ ovarian^56^ or endometrial^57^, ^58^ but also in other cancers, including CRC and lung cancer.^59^, ^60^, ^61^, ^62^, ^63^ It regulates cellular mechanisms such as cell cycle, the stress of the endoplasmic reticulum and apoptosis in colon cancer cells.^28^ Activation of GPER by G‐1 ligands results in cell cycle arrest and inhibition of proliferation in colon cancer cells.^28^ Accumulation of colon cancer cells in the apoptotic sub‐G1 phase and lower mitochondrial membrane polarity followed by GPER activation suggested GPER mediated apoptosis of colon cancer cells. Thus, activation of GPER‐mediated signaling plays crucial roles in the proliferation and survival of colon cancer cells.

Endoplasmic reticulum stress, another crucial regulatory phenomenon of growth arrest and apoptosis of cancer cells, is associated with GPER mediated signaling.^64^ Liu et al. reported that expression of several important regulators of endoplasmic reticulum stress such as activating transcription factor 4 (ATF4), activating transcription factor 6 (ATF6), X box‐binding protein 1 (XBP‐1)and C/EBP‐homologous protein (CHOP)was increased, followed by G‐1 treatment in colon cancer (HCT116) cells.^28^ Furthermore, GPER mediated signaling caused elevated generations of reactive oxygen species (ROS) and increased ERK1/2 phosphorylation, which subsequently contributed to the growth arrest of colon cancer cells.^28^

Additionally, GPER activation affects other cellular behaviors such as migration and invasion in multiple cancer types.^60^, ^65^, ^66^, ^67^, ^68^, ^69^, ^70^ Importantly, activation of GPER plays dual roles on (HT‐29 and DLD‐1) colon cancer cells' invasion and migration.^67^ In normoxic conditions, both G‐1 and estrogen inhibited the migration of cancer cells, whereas, in hypoxic conditions, GPER stimulation increased the migration of colon cancer cells.^67^ Another estrogen‐regulated oncogene, namely Fatty acid synthase (FASN) acts as a key lipogenic enzyme, affects neoplastic transformation of the cells in the breast, colon and liver.^29^, ^71^, ^72^, ^73^ GPER induced elevated expression and activity of FASN through EGFR/ERK/c‐Fos/AP‐1 signaling, which subsequently resulted in increased growth and migration of colon cancer cells.^29^ Thus, GPER activity regulates cell motility through multiple complex mechanisms. Down‐regulation of GPER in mRNA and protein level was seen in cancer tissue samples compared to adjacent non‐neoplastic tissues.^28^ The downregulation of GPER in CRC patients associated with lymph node metastasis, tumor progression and poorer survival rates of patients with cancers.^28^, ^64^ Also, epigenetic changes were responsible for GPER downregulation in CRC compared to their adjacent non‐neoplastic tissues.^28^ Two of the vital epigenetic modulations, i.e. promoter methylation and histone H3 deacetylation, were associated with the downregulation of GPER in both in‐vitro and in‐vivo in CRCs.^28^

There are some controversies that exist regarding the role of GPER, whether it acts as a tumor suppressor or a tumor promoter in CRC development.^20^, ^30^ Increased GPER expression was noted in colon cancer cells followed by Nonylphenol (NP), an endocrine disruptor (found in cleaners, plastics, and detergents) treatment, which in turn induces the proliferation of colon cancer cells and inhibited apoptosis in vitro.^74^ Upregulation of Cyclin D1, p‐PKA, c‐Myc, and proteins that regulate the cell cycle was occurred, followed by NP treatment in colon cancer cells.^74^ Treatment of cells with G15, a GPER inhibitor, abrogated the NP mediated ERK1/2 activation and subsequent cell proliferation.^74^ On the other hand, an in vivo study reported that mice receiving G15 treatment had impaired tumor growth.^75^ The study further confirmed GPER activation by its specific agonist G‐1 resulted in decreased proliferation, enhanced cell cycle arrest, mitochondrial apoptosis and endoplasmic reticulum stress in colon cancer cells.^75^ Taken together, it can be suggested that further studies are warranted to confirm the role of GPER mediated signaling in colon cancer cells.

## 3 NATURAL COMPOUNDS SUPPRESSING CRC TUMOURIGENESIS THROUGH ACTING AS AN ER AGONIST

Selective ER agonists could have the potential to stimulate the tumor suppressor function of ER in CRC. A number of both natural and synthetic ligands for ERβ have shown to play protective roles against CRC development (Table 3). Phytoestrogens and xenoestrogens bind to estrogen receptors preferentially due to their structural similarity to estrogen.^27^ Following binding, they generate similar or varying effects on cancer cell proliferation and progression depending on the structure of the compounds. Xenoestrogens are the synthetic counterpart of estrogens, varying in structure and activities. These synthetic chemicals are potentially harmful and could predispose human to adverse health effects.^81^ On the other hand, phytoestrogens are plant‐derived compounds that may have protective roles against CRC development, followed by biding to ERβ.^82^ Pre‐clinical studies suggested that plant‐derived flavonoids play protective roles against endocrine associated cancers, including CRC.^76^, ^83^, ^84^ Diet enriched with various phytoestrogens inhibited the generation of polyps and cryptic adenomas in ovariectomized Apc Min/+ female mice. Moreover, phytoestrogens containing diet dose‐dependently inhibited azoxymethane‐induced intestinal carcinogenesis by elevating the expression of ERβ in the colonic mucosa.^85^, ^86^ Another study using female ER‐β−/− mice reported that dietary fiber in combination with estrogenic isoflavones might effectively alter the gut microbiota through ERβ, which in turn modifies local inflammatory processes to prevent colonic tumorigenesis.^87^ The roles of a few natural and synthetic ERβ agonist compounds in the inhibition of CRC tumourigenesis is illustrated below.

**TABLE 3:** Natural or synthetic compounds that inhibit colon cancer cells through estrogen receptor signaling

Compounds	Source	Functions	References
Genistein	Soya product	Acts as an ERβ‐specific agonist and exerts anti‐proliferative and pro‐apoptotic effects by reducing the expression of oncogene p63, PCNA expression and by increasing expression of cleaved cytokeratin‐18.	[76]
Quercetin	Leafy vegetables, broccoli, red onions etc.	Causes cell cycle arrest, increases apoptosis, modulates estrogen receptors, regulates signaling pathways, inhibits metastasis and angiogenesis etc. in CRC by binding to cannabinoid CB1 receptor	[77]
Calycosin	*Radix astragali*	Increases the expression of ERβ and reduces the expression of ERα, insulin‐like growth factor receptor (IGF‐1R), and p‐Akt. Also causes down‐regulation of miR‐95 to increase the apoptosis of colon cancer cells.	[78]
17β‐estradiol	Endogenous	Activates p53 signaling, which results in up‐regulation of p21 and p27 proteins.	[79]
Diarylpropionitrile (ERβ ligand)	Synthetic compound	Up‐regulation of p21 and p27 proteins resulted in inhibition of their target gene cyclin D1 to inhibit cell proliferation	[79]
Propylpyrazole‐triol (ERα ligand)	Synthetic compound	Inhibits cell migration by decreasing the expression of urokinase plasminogen activator (u‐PA), tissue‐type plasminogen activator (t‐PA) and matrix metalloproteinase 9 (MMP‐9) as well as matrix metalloproteinase 2/9 (MMP‐2/9) activity in colon cancer cells.	[79]
Silibinin	*Silybum marianum*	Induces cell cycle arrest and apoptosis in vitro in colon cancer cells	[80]

### 3.1 Genistein

Genistein, one of the isoflavones derived from soy product, has been reported to inhibit proliferation and promotes apoptosis by increasing the expression of ERβ followed by activation of various pathways in cancer cells.^76^ In addition, ovariectomized rats exhibited a significant increase in apoptosis and a reduction in the proliferation of colonocytes, followed by Genistein treatment.^84^

### 3.2 Quercetin

Quercetin, a natural flavonol found in commonly consumed food items, has been demonstrated inhibitory effects on cancer progression by multiple mechanisms.^77^, ^88^ Diets containing Quercetin, such as vegetables, fruits, and grains, associated with a reduced risk of colon cancer, which indicated the anti‐cancer potential of quercetin in CRC.^89^, ^90^ Quercetin restrains the expression of cellular estrogen‐responsive receptors such as cannabinoid CB1 receptor (CB1‐R), a G‐protein‐coupled cannabinoid receptor associated with the proliferation of colon cancer cell.^91^ Quercetin binds to CB1‐R and regulates cell growth rate in Caco2 and DLD‐1 colon cancer cells. Quercetin showed its anti‐cancer effects by causing cell cycle arrest, increasing apoptosis, modulating estrogen receptors, regulating growth signaling, and inhibiting metastasis etc., in CRC.^92^ Quercetin is structurally similar to estrogen, therefore, can effectively bind to the CB1‐R, and thereby regulate CRC cell growth and proliferation. A significant increase in the expression levels of CB1‐R was noted, followed by quercetin treatment; however, this induction was partially abolished with the use of a specific CB1‐R antagonist. These results indicate CB1‐R dependent action of quercetin in CRC.

### 3.3 Calycosin

Calycosin, another plant‐derived isoflavone, inhibited the proliferation and induced apoptosis of colon cancer (SW480 and LoVo) cells.^78^ Calycosin increased the expression of ERβ and reduced the expression of ERα, insulin‐like growth factor receptor (IGF‐1R), and p‐Akt. It also caused the down‐regulation of miR‐95 in cancer cells. It was noted that inhibiting ERβ confined the change of miR‐95 expression, which subsequently resulted in increased apoptosis of cancer cells. Furthermore, calycosin significantly inhibited xenograft tumor growth in nude mice model.^78^

### 3.4 Diarylpropionitrile (DPN)

Estradiol (17β‐estradiol) such as diarylpropionitrile (DPN), a selective agonist of ERβ exhibited anti‐cancer properties against colon cancer cells.^79^ Hsu et al. reported that treatment of cancer cells with these compound activated p53, which in turn activated p21 and p27 proteins and their downstream signaling. Activation of those cell cycle proteins subsequently reduces the expression of cyclin D1, which ultimately inhibits cell proliferation.^79^ Also, 17β‐estradiol and ERβ agonists (DPN) significantly inhibited the expression of urokinase plasminogen activator (u‐PA), tissue‐type plasminogen activator (t‐PA), matrix metalloproteinase 9 (MMP‐9) and matrix metalloproteinase 2/9 (MMP‐2/9), which in turn inhibited colon cancer cells invasion, migration and metastasis. Pretreatment colon cancer (LoVo) cells with a p53 inhibitor significantly restrained the anti‐migration effects of ERβ agonists.^79^ Thus, 17β‐estradiol and/or ERβ agonists may have the potential to be used as potent alternative therapeutics in the treatment of human CRC.

### 3.5 Silibinin

Silibinin, a flavonolignan extracted from *Silybum marianum*, has strong cancer chemo‐preventive properties against various cancers, including colon cancer.^93^, ^94^, ^95^, ^96^, ^97^, ^98^, ^99^ Agarwal et al. suggested that this compound induced cell cycle arrest and apoptosis in vitro in human colon cells.^93^ In addition, oral supplementation of silibin into nude mice suppressed HT‐29 xenograft growth by inhibiting cell proliferation and angiogenesis.^80^ Also, Silibinin showed cancer‐protective activities by preventing azoxymethane (AOM)‐induced colonic aberrant crypt foci formation in Fisher 344 rats.^100^ However, the underlying mechanism of silibinin in the ER‐mediated cancer response needs further investigation. In addition, a clinical trial using natural compounds as ER agonist (Eviendep) was tested to examine the recurrence of adenocarcinoma of colon (NCT01402648). Taken together, targeting estrogen and its receptors with synthetic or natural compounds shows promising opportunities for better management of patients with CRCs.

## 4 CONCLUSION

This review provides a detail of the current literature on the association of estrogen and estrogen receptors in CRC carcinogenesis and potentiates some compounds to be used to enhance the anti‐tumourigenic activity of ERs. Further studies of the estrogen‐signaling pathway may increase the knowledge of the underlying mechanism of ER driven protection of CRC pathogenesis. For example, chronic inflammation of the colon (colitis) induced CRC pathogenesis is protected by intestinal epithelium ERβ expression, whereas loss of ERβ expression promoted carcinogenesis in the colorectum.^101^ In addition, intestinal ERβ expression prevent the development of colitis‐associated CRC by regulating the expression of TNFα, a regulator of NFκB signaling network.^102^ Thus, modulation of ERs have roles in developing potential anti‐cancer drugs in CRCs. Therefore, upregulation of ERβ with specific ligands such as phytoestrogens may help to confer a protective effect in the colon by repressing oncogenic transformation and metastasis.

## AUTHOR CONTRIBUTIONS

**Plabon Kumar Das:** Data curation (lead); writing – original draft (lead). **Joti Saha:** Data curation (supporting). **Suja Pillai:** Formal analysis (supporting); writing – review and editing (supporting). **Alfred K.‐Y. Lam:** Writing – review and editing (supporting). **Vinod Gopalan:** Conceptualization (supporting); writing – review and editing (supporting). **Farhadul Islam:** Conceptualization (lead); supervision (lead); writing – review and editing (lead).

## FUNDING INFORMATION

This research did not receive any specific grant from funding agencies in the public, commercial, or not‐for‐profit sectors.

## CONFLICT OF INTEREST

The authors declare no conflict of interest.

## ETHICS STATEMENT

Not applicable.

## Data Availability

Data included in article/supplementary material/referenced in the article.
